# AAWS-Net: Anatomy-aware weakly-supervised learning network for breast mass segmentation

**DOI:** 10.1371/journal.pone.0256830

**Published:** 2021-08-30

**Authors:** Yeheng Sun, Yule Ji

**Affiliations:** School of Business, University of Shanghai for Science and Technology, Shanghai, China; Newcastle University, UNITED KINGDOM

## Abstract

Accurate segmentation of breast masses is an essential step in computer aided diagnosis of breast cancer. The scarcity of annotated training data greatly hinders the model’s generalization ability, especially for the deep learning based methods. However, high-quality image-level annotations are time-consuming and cumbersome in medical image analysis scenarios. In addition, a large amount of weak annotations is under-utilized which comprise common anatomy features. To this end, inspired by teacher-student networks, we propose an Anatomy-Aware Weakly-Supervised learning Network (AAWS-Net) for extracting useful information from mammograms with weak annotations for efficient and accurate breast mass segmentation. Specifically, we adopt a weakly-supervised learning strategy in the Teacher to extract anatomy structure from mammograms with weak annotations by reconstructing the original image. Besides, knowledge distillation is used to suggest morphological differences between benign and malignant masses. Moreover, the prior knowledge learned from the Teacher is introduced to the Student in an end-to-end way, which improves the ability of the student network to locate and segment masses. Experiments on CBIS-DDSM have shown that our method yields promising performance compared with state-of-the-art alternative models for breast mass segmentation in terms of segmentation accuracy and IoU.

## Introduction

Breast cancer is one of the common cancer types that seriously endangers women’s health around the world [1]. Early diagnosis, effective prediction and collaborative healthcare can significantly reduce the mortality rate of breast cancer [2–4]. Mammography screening becomes the most reliable tool for detecting breast carcinomas at early stages, because it provides an informative, non-invasive and inexpensive way of examination [5,6]. However, it is extraordinarily difficult to detect masses even for experienced radiologists, since their high variability, low contrast and high similarity with the surrounding healthy tissues [7]. Therefore, the X-ray Computer Aided Diagnosis (CAD) of abnormalities is highly recommended for assisting radiologists in locating masses and outlining borders of masses. In particular, accurate semantic segmentation of breast mass plays a key role in feature extraction and related downstream procedures [8].

In common sense, the early state-of-the-art architectures generally benefit from well hand-designed feature representations (e.g., intensity information, contrast information and texture information, etc.) [9–11]. Those conventional medical segmentation techniques excessively depend on the prior knowledge of medical science and subjective assessment which hinders its widespread application in medical image analysis. Despite the early popularity and success of the traditional segmentation methods, Deep Learning (DL) approaches change the main trend on many segmentation tasks in the context of medical image analysis (e.g., semantic segmentation). In medical image segmentation scenarios, semantic segmentation classifies each pixel of a medical image and provides both location and rich morphology features, such as shape type (round, oval, lobular and irregular) and margin type (spiculate, ill-defined, obscured and circumscribed) [12]. DL based algorithms are the data-driven machine learning paradigm, which has an intrinsic ability to learn deep and discriminative features from data without human intervention, and thence, outperform the traditional image segmentation methods [13]. Previously, DL based solutions segmented abnormities by sliding the image block with fixed-size which is intercepted by a square window centered on the target pixel. Whereas, the segmentation accuracy depends on the size of the selected image block and the computation time increases due to repeated computations [14].

After the advent of Fully Convolutional Networks (FCN), such brand-new network structures gradually replaced the sliding window algorithm and became the mainstream framework for semantic segmentation [15]. FCN adopts a classic encoder-decoder structure, replaces the full connection layer in Convolutional Neural Networks (CNN) with the convolutional layer and uses the transpose convolution to perform accurate segmentation. Based on FCN, the difference of various segmentation networks is mainly manifested in the construction of decoder [16,17]. Although FCN achieves accurate semantic segmentation by multi-scale feature fusion, the problem of inaccurate pixel positioning affects the accuracy of segmentation [18]. U-Net is a commonly used network for medical image segmentation, which adopts a completely symmetrical encoder-decoder skeleton with skip connections. In this way, it can fuse high- and low-level information and produce more accurate pixel positioning by concatenation, thus achieves higher segmentation accuracy [19]. Derived from U-Net, a series of state-of-the-art networks for medical image segmentation were proposed, most of which benefit from attention mechanism [20], recurrent residual convolution [21], residual connection [22], to name a few. For instance, deep supervision was integrated with U-Net to improve U-Net’s attention to the boundary of Region of Interests (ROIs) [23]. Furthermore, a modified U-Net coupled with Attention Gates (AGs) for breast mass segmentation was proposed in [24]. In this method, U-Net adopts a densely connected convolutional network as an encoder and AGs as the decoder network. In another study [25], a multi-task model by combining both the residual connections and deep supervision scheme with U-Net was presented. Another approach is UNet++ which uses a shallow structure adapted from the original U-Net, thus avoiding loss of details when transferring information from encoder to decoder, which is great of importance for accurate boundaries detection [26,27]. While UNet 3+ combines deep and shallow features through full-scale connection and supervises the features of each scale by deep supervision [28].

Although the architectural advancements have achieved outstanding performance on the semantic segmentation task of medical images, they still share the common limitations for further improvements, i.e., both large and high-quality annotated datasets are needed for training an efficient image segmentation model [29]. However, the acquirement of numerous annotated datasets remains a tricky challenge, especially in medical image processing, where annotating mass instances requires experts to revisit mammograms and outline the edges of tumor, which is extremely cumbersome and time-consuming [30]. Although potential feasible solutions such as crowdsourcing could generate large amounts of images with accurate annotations undesired noise would be incurred [31]. To leverage numerous medical images with weak annotations, weakly-supervised learning strategies have been developed to excavate specific types of information from data with scarce annotations or weak annotations (e.g., sparse annotations, noisy annotations and bounding box annotations) [32–34]. Besides, Knowledge Distillation (KD) can offset the deficiency of annotations by transferring prior information [35,36]. Moreover, the current state-of-the-art methods are commonly committed to exploring important information from limited data for per-pixel analysis, while under-utilize numerous medical images with scarce annotations or weak annotations. Furthermore, both labeled and unlabeled datasets abundantly contain the same anatomical structures which commonly share the same properties because of the inherent nature of anatomy. Anatomy features can guide abnormalities segmentation by comparison with the same features existing in other un-labeled images [37]. Therefore, a full exploration of anatomical structures in medical images may greatly enhance the segmentation performance of DL based models [38,39]. On top of that, clinical data show that the morphological features of mass are highly related to tumor grade (i.e., benign and malignant), which is an important reference basis for clinicians to make the diagnosis and further give treatment options [40]. Generally speaking, benign tumors present regular shapes, while malignant tumors usually have irregular borders. However, in some cases, it is difficult to tell whether a mass is benign or malignant [41]. Therefore, the accurate classification of the mass is of great importance.

To this end, we propose an Anatomy-Aware Weakly-Supervised Learning Network (AAWS-Net) to perform breast mass segmentation. Specifically, a well-trained Auto-Encoder (AE) is employed to explore common anatomical structures from large sets of mammographic images with weak annotations. Moreover, to push forward segmentation performance, KD is introduced to transfer anatomy features representation ability from the AE (Teacher) to the segmentation network, i.e., U-Net (Student) so that the segmentation network becomes more sensitive to other anatomy structures when the available data is limited. Furthermore, to further boost the segmentation performance, we present a scheme of mass classification which can suggest the morphological differences between benign and malignant masses for the network and provide the reference basis for the therapeutic plan.

To sum up, the main contributions of this paper are four-fold:

- We proposed a systematic weakly-supervised learning framework which is an end-to-end trainable network for breast mass segmentation. The proposed model could make full use of weak annotations.
- Common anatomical structures of breast were fully explored by reconstructing the original image.
- KD was introduced to distinguish the morphological differences between benign and malignant masses to further enhance the segmentation ability.
- The proposed network was evaluated and compared with the other four methods for mass segmentation of mammograms on the CBIS-DDSM dataset, showing that it overwhelms the others.

The rest of this paper is structured as follows in a whole. First, the employed methodology and experiment are illustrated in Section 2. Then, the achieved results are presented in Section 3. Furthermore, the experimental conclusion and future work are provided in Section 4.

## Materials & methods

In this section, the proposed network architecture, including the Teacher model and the Student model are presented. After that, the details of experiments are illustrated. Finally, quantitative evaluation metrics are listed.

### Anatomy-Aware Self-Supervised Learning Net (AAWS-Net)

The goal of the proposed framework is to boost the accuracy of breast mass segmentation model by fully utilizing large-scale weakly-labeled dataset and exploiting the breast anatomy structures of mammograms. To further improve the performance of the proposed model, KD was introduced for classifying the pathology of masses. In particular, AE (Teacher) was designed for better representation of anatomy structures, while traditional U-Net (Student) was performed for accurate segmentation of breast masses.

**Fig 1:**
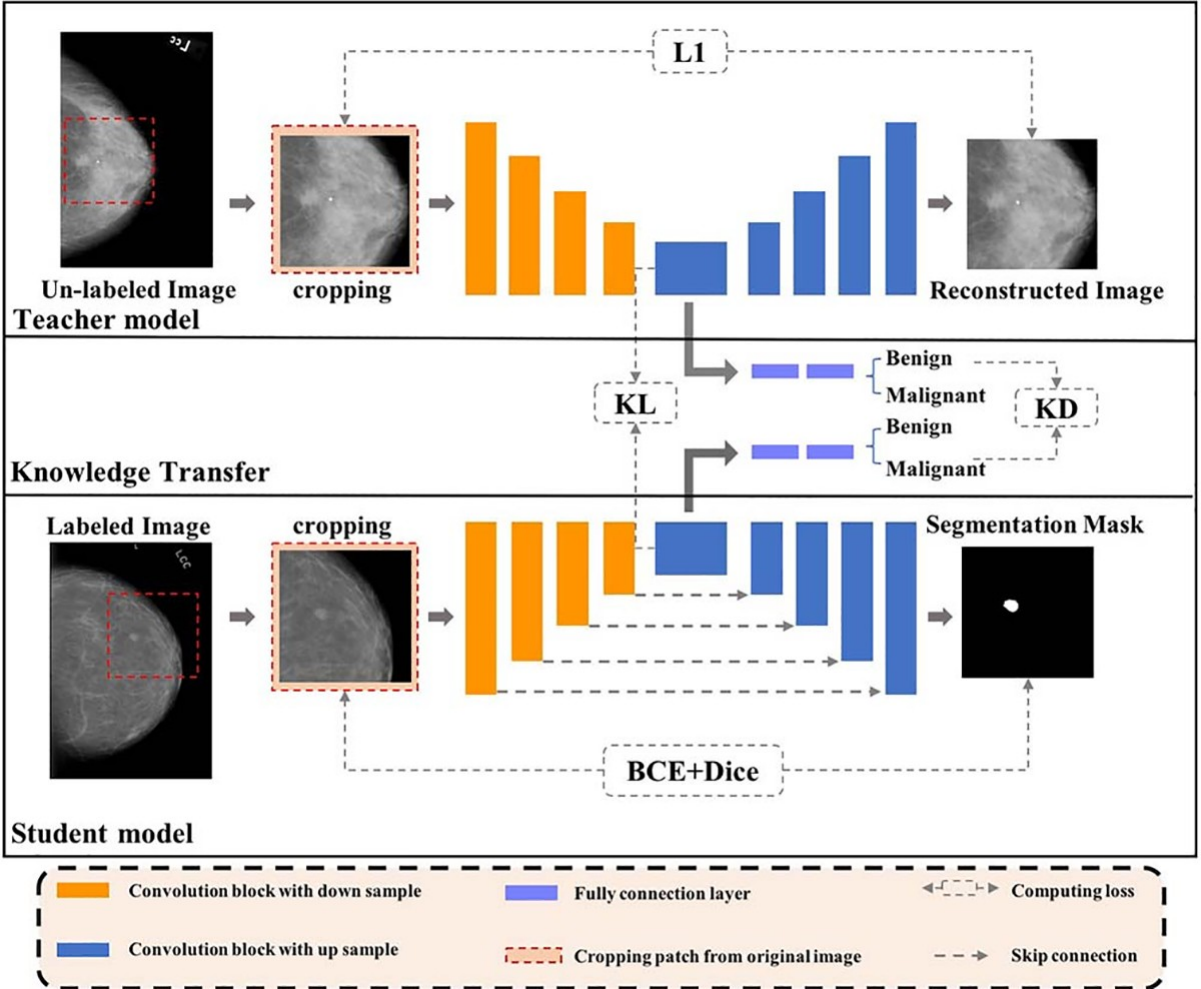
Illustration of AAWS-Net.

As is shown in Fig 1, both the Teacher and the Student have the same symmetric encoder-decoder architecture adapted from the version of U-Net in [19], and the proposed framework is constructed by three branches. The first branch enforces a weakly-supervised learning network (i.e., a symmetric encoder-decoder without skip connections) to learn anatomical structures of the breast by reproducing the original input image on the dataset without corresponding binary masks. Then, KL loss was used to transfer anatomy properties of the Teacher to the Student for promoting the accuracy of mass segmentation. Furthermore, a classification scheme of masses was embedded in the framework, which could provide clues of the difference between benign and malignant masses for the segmentation network through the application of Knowledge Distillation (KD) loss. The third stage adopts the Ground Truth (GT) loss to make the Student’s prediction of segmentation similar to that of the reference segmentation. Note that the Teacher and Student have the same number of encoder and decoder nodes. In addition, the weights of the Teacher will not be updated when training the Student. The input image is a squared crop of the full mammogram containing abnormalities.

#### Teacher model

In the medical image domain, due to relatively stable structure of organs, uncomplicated and unequivocal semantic information contained in images, as well as fuzzy boundaries and complicated gradients, thus both high- and low-level semantic information are of great importance for accurate segmentation.

**Fig 2:**
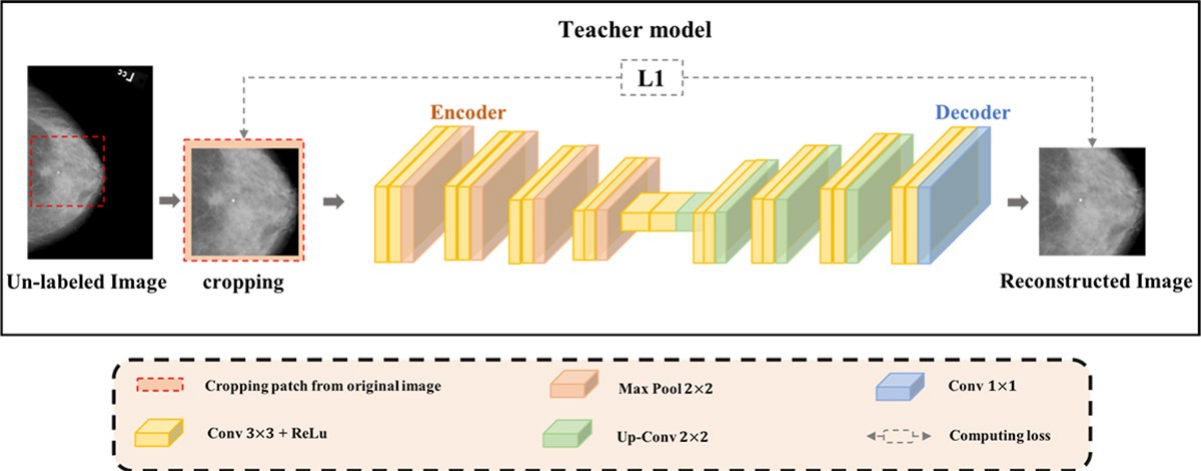
Teacher network architecture.

In this study, the Teacher network is a well-known self-supervised learning network, i.e., AE, which is a common feature compression scheme, encoding high-dimensional input data into low-dimensional output codes and then reconstructing the original input from codes. Recent studies have demonstrated the efficiency of medical image segmentation on limited datasets with weak annotations [34]. To be specific, the encoder network aims to compress high-dimensional input properties into low-dimensional output features, while the symmetric decoder network then upsamples the low-resolution encoder feature maps to full input resolution features to reconstruct the original input. The purpose of the Teacher is to learn anatomy features by restoring input image tile in latent space. Fig 2 shows the detailed architecture of the Teacher.

As we can see from Fig 2, the encoder of the Teacher obtains low-resolution feature maps by down-sampling four times, which can provide contextual semantic information of the segmented object in the whole image. More importantly, the contraction path can study anatomy features by encoding them at different levels. Symmetrically, the decoder reverts the low-resolution semantic features to the high-resolution of the original image by upsampling four times. This process can extract both high- and low-level information, thus improve the feature representation capability of the Teacher.

#### Student model

**Fig 3:**
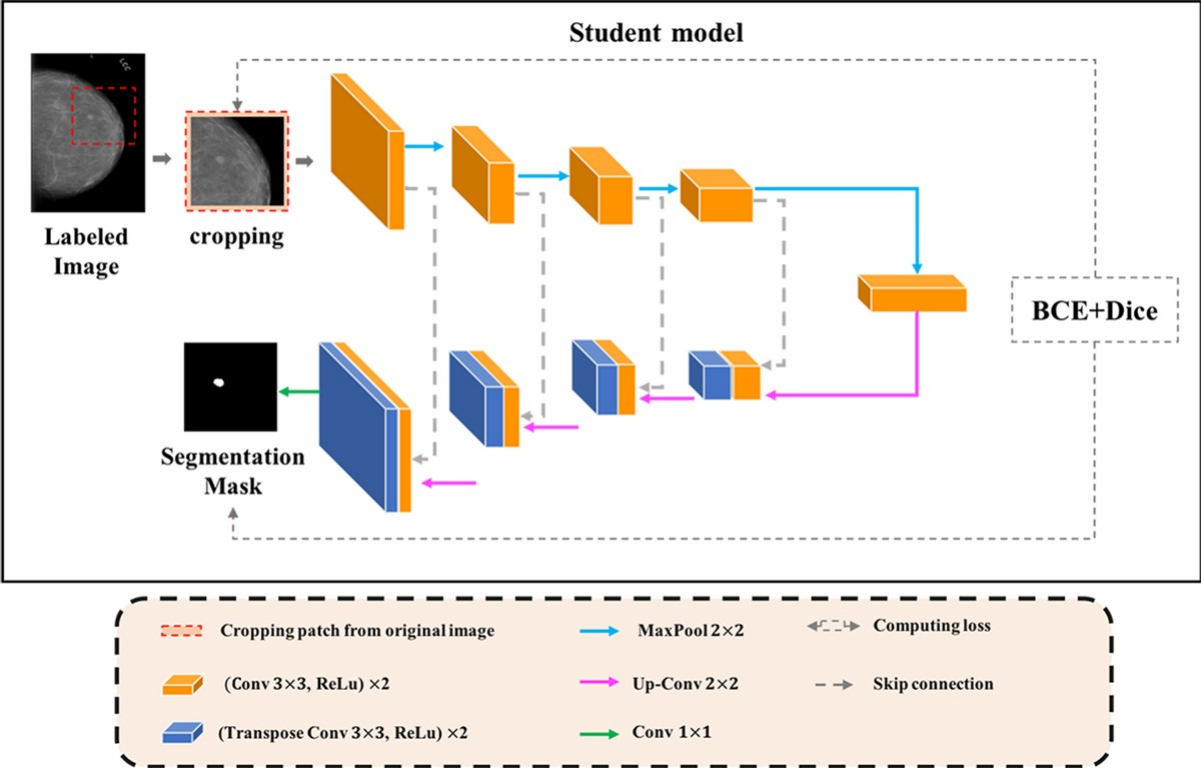
Backbone architecture of the student.

The Student adopts conventional U-Net in [19] (Fig 3) as backbone network, which is composed of two main paths (i.e., down-sampling and up-sampling) and a transition block is at the bottom of the network. Different from the Teacher, the Student adopts skip connections at the same stage of the corresponding encoder and decoder phase. Thus, the segmentation feature map fussed not only low-level features, but also features at different scales, which can improve the accuracy of the segmentation model and prevent gradients from vanishing. Moreover, four times of upsampling also makes the segmentation image recover edge information more refined.

The Student learns privileged information from the Teacher by adding the soft targets of the Teacher, which are shown as follows:

$$\rho_{i}=\frac{exp(\tau_{i}/T)}{\sum_{j}exp(\tau_{i}/T)}$$ (1)

Where *T* is a parameter used to control the importance of the soft targets. Given the logarithmic vector *τ* as the output of the last full connection layer, *τ*_*i*_ is the log of class *i*.

As we can see from Fig 1, the Teacher and Student process different inputs and have different goals. Specifically, the Teacher aims at exploring breast anatomical structures by fully leveraging weakly-annotated data, while the Student serves for learning the transferred knowledge from the Teacher to obtain accurate semantic segmentation of mammographic images. In consequence, we transfer the Teacher’s anatomy features representation capability to the Student in latent feature space.

#### Knowledge transfer

Owing to different inputs of Teacher and Student, low-level image information (e.g., texture, edges and gradients) encoded in the lowest layers should be different. By contrast, higher-level image properties in the highest layers closer to the bottleneck should be similar. Moreover, we assume that the Teacher encodes the anatomical properties to precisely reconstruct the original images [42]. Then, we adopt KD as knowledge transformation strategy to transfer the anatomy information encoded in the Teacher’s bottleneck to the Student by making their anatomy features representation capabilities as close as possible.

In particular, we use KL loss to compare the dissimilarity between the latent spaces which allows the Student to obtain anatomy features representation ability from the Teacher. Besides, the KD loss term enables the Student network to learn specific information from the Teacher by classifying the type of breast mass via image-wise labels.

#### Training strategy

At first, we train a weakly-supervised learning network (i.e., AE) on a large-volume of weakly-labeled datasets to directly learn the anatomy features of breast masses by encouraging the Teacher to reconstruct the original image. Then, we transfer privileged knowledge from the pre-trained Teacher to Student by distillation. In particular, weights of the Teacher will not update by backpropagation and three different losses (i.e., KL loss, KD loss, and GT loss) are used when training the Student.

#### Loss function

Reasonable selection of loss function is of great importance in improving the performance of models [43]. In order to encourage the Teacher to learn high-level semantic properties of anatomical structure, rather than pixel-wise features, we used L1 loss for the restoration task. At the same time, we employed Cross Entropy (CE) loss to make the Teacher model classify the pathologic types of breast mass from the latent features.

$$L_{L1}=\frac{1}{N}\sum_{i=1}^{N}\left|y_{i}-{\hat{y}}_{i}\right|$$ (2)

$$L_{CE}=-z\;\log(\hat{z})-(1-z)\log\left(1-\hat{z}\right)$$ (3)

Where ${\hat{y}}_{i}$ and *y*_*i*_ represent the *i*^*th*^ pixel value of the reconstruction image and the input image respectively. *N* is the total number of pixels in the image. $\hat{z}$ denotes the probability that the sample is predicted to be positive, and *z* is the corresponding label of the sample.

The total loss of the Teacher is expressed by:

$$L_{T}=L_{L1}+L_{CE}$$ (4)

Based on that, KL loss can measure the difference between predicted image and labeled image, which was used for transferring useful privilege information of anatomical structures encoded in the Teacher’s bottlenecks to the Student’s ones.

$$L_{KL}(p,q)=\sum_{i}\sum_{j}\sum_{j}q_{i}(j)\log\left(\frac{q_{i}(j)}{p_{i}(j)}\right)$$ (5)

Where *p*_*i*_ (resp. *q*_*i*_) denotes the bottleneck’s flattened and normalized vector. We use KL loss to encourage the distribution of the Student’s bottleneck to be similar to the one of the Teacher’s.

KD loss was applied for pointing out the difference between benign and malignant masses to further enhancement of segmentation accuracy.

$$L_{KD}=\sum_{i}\left[\left(1-Dice(\rho_{i},\sigma (\tau_{s}^{i})))+BCE(\rho_{i}^{*},\sigma (\tau_{s}^{i})\right)\right]$$ (6)

Where *σ* is the softmax function, $\rho_{i}^{*}$ is the binary prediction of the Teacher. *Dice* is the Dice score, which measures the similarity of feature maps of the Teacher and Student. *BCE* is the binary cross entropy, which is a local measure computed for each pixel. Note that it is necessary to binarize *ρ*_*i*_ since soft labels cannot be used in the binary cross entropy.

Additionally, we enforce the Student to generate the segmentation prediction as close as possible to the ground truth by using the reference segmentation loss function, which is computed as follows:

$$L_{GT}=\sum_{i}\left[\left(1-Dice(s_{i},\sigma (\tau_{s}^{i})))+BCE(s_{i},\sigma (\tau_{s}^{i})\right)\right]$$ (7)

Where *s*_*i*_ represents the *i*^*th*^ sample’s corresponding ground truth mask in the dataset.

The complete objective function of the Student is:

$$L_{S}=\alpha L_{KD}+(1-\alpha )L_{GT}+\lambda L_{KL}$$ (8)

Where, *α*∈[0, 1],*λ*∈ℝ^+^. The parameter *α* balances the influence of the Student’s hard targets with the Teacher’s soft targets, while *λ* is used to balance the magnitude of the KL loss with the other two losses.

### Experimentation

#### Dataset of mammographic images

We validated AAWS-Net on a publicly available digital dataset for screening mammography: CBIS-DDSM (Curated Breast Imaging Subset of DDSM) [44,45] which contains 891 mass cases. In this work, a total of 1250 images in CC view which contains full mammogram images, cropped ROI and corresponding ground truth of binary masks were chosen for mass segmentation. We used 80% of the images for training, 10% for validation and the rest for testing the breast mass segmentation model. All of the full mammogram images as well as the related masks were first converted into PNG format and then cropped to 1024×1024 to remove the irrelevant background regions, followed by flipping and intensity normalization.

#### Experiment setting

In our experiments, we evaluate the segmentation performance of AAWS-Net and four deep models of recent segmentation references (i.e., U-Net [19], FCN [15], ResUnet [22] and DenseUnet [46]).

To further validate our proposed network’s segmentation performance, the ablation experiments were carried out according to the strategy in [47]. First of all, traditional U-Net was used for mass segmentation on CBIS-DDSM with pixel-wise masks. Afterward, AE (i.e., a symmetric encoder-decoder without skip connections) explored anatomical structures on the same datasets but the pixel-level annotations were removed, then the pre-trained AE was applied for the mass segmentation task. Likewise, U-Net was employed for reconstruction and segmentation tasks. In addition, in order to further investigate the effects of anatomical structures and mass classification on boosting segmentation accuracy, the proposed framework first abandoned KD loss but retained the KL loss. Then, KL loss was used rather than KD loss, and finally, both KD loss and KL loss were adopted for mass segmentation.

All the models shared the same setting and training strategies, and to save the training time, an early-stopping mechanism was also applied during training. All of experiments were conducted by python with pytorch1.1.0, torchvision0.3.0 and cudatoolkit9.0 performing on a 64-bit Ubuntu operating system using a 2.9 GHz Intel Core-i5 with 15.6 GB of RAM and Nvidia GTX 1080ti GPU with 11 GB of video RAM.

### Evaluation metrics

In this study, we used five metrics for model evaluation: accuracy (AC), recall (RE), F1 score (F1), specificity (SP) and Intersection over Union (IoU), which are computed as follow:

$$AC=\frac{TP+TN}{TP+FP+TN+FN}$$ (9)

$$RE=\frac{TP}{TP+FN}$$ (10)

$$F1=2\times \frac{recall\times precision}{\left(recall+precision\right)}$$ (11)

$$SP=\frac{TN}{FP+TN}$$ (12)

$$IoU=\frac{target\cap prediction}{target\cup prediciton}$$ (13)

Where TP, FP, TN and FN refer to true positives, false positives, true negatives and false negatives, respectively.

## Results and discussion

### Segmentation experiment results and discussion

In this section, segmentation results in terms of four crucial metrics used in our experiment of all models (i.e., AAWS-Net, U-Net, FCN, DenseUnet and ResUnet) are summarized in Table 1.

**Table 1:** The comparison of segmentation results.

Datasets	Methods	IoU	AC	SP	F1
CBIS-DDSM	U-Net	0.628	**0.962**	**0.854**	0.738
	FCN	0.599	0.932	0.782	0.726
	DenseUnet	0.649	0.946	0.793	0.758
	ResUnet	0.644	0.937	0.807	0.755
	AAWS-Net	**0.672**	**0.962**	0.824	**0.791**

As is shown in Table 1, the best results are marked in bold and AAWS-Net yields the best results in comparison with the other four methods across all metrics except for the SP value. U-Net obtains the best result of 85.4% while our proposed model provides the second-best result of 82.4% with a modest difference of 3%. In the IoU column, AAWS-Net outperforms the compared methods, where DenseUnet is the second best model. Contrasted to U-Net, AAWS-Net obtains the same degree of accuracy, which is 96.2%. Although the specificity of AAWS-Net (82.4%) is not as good as U-Net (85.4%), the former has obtained an impressive F1(79.1%), which leads to the highest values of IoU (67.2%). The U-Net model obtains the highest rate of true negatives, i.e., SP (85.4%) and AC (96.2%) but its F1 score is poorer (73.8%) than AAWS-Net, which informs that it misses more real tumor area than the AAWS-Net approach.

**Fig 4:**
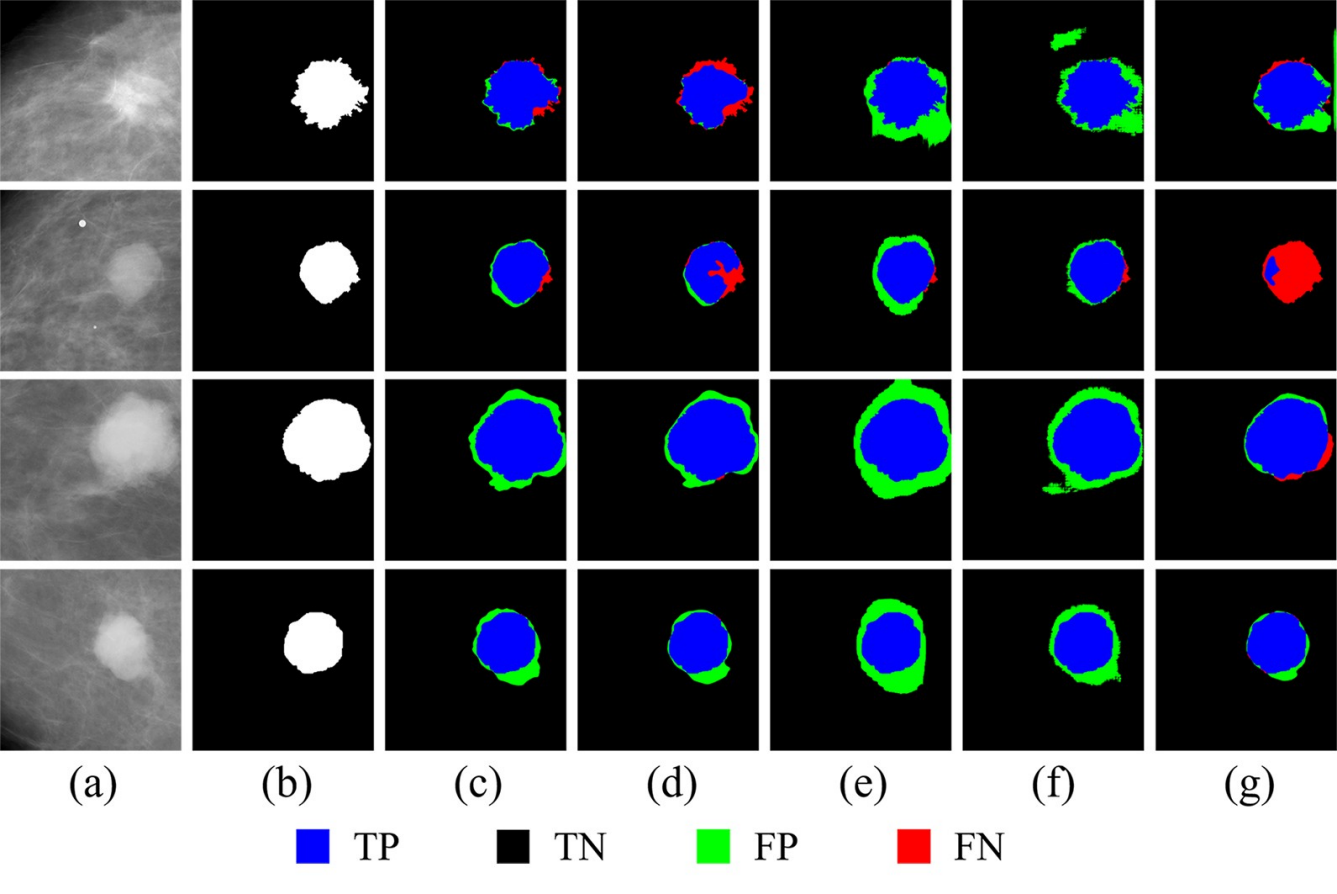
Segmentation results of five models with CBIS-DDSM dataset.

In general, our proposed model performed well in terms of crucial metrics which supports our hypothesis that the fully-utilized weakly-annotated data and fully-exploited anatomy features provide more details of breast mass and support and play a key role in accurate mass segmentation. Fig 4 presents a visualization of four tumors’ segmentation results by five segmentation approaches on CBIS-DDSM dataset. To be specific, cropped original images and corresponding ground truth masks are shown in the first and second column, respectively, and the TP, TN, FN, and FP are marked up in red, black, blue and green.

Fig 4 provides a comparison of the segmentation results between the proposed model and the other five methods on CBIS-DDSM. Columns (a) and (b) are cropped original images and corresponding ground truth masks, respectively. Columns (c)-(g) are the segmentation results from AAWS-Net, U-Net, DenseUnet, FCN, and ResUnet, respectively. It can be seen from Fig 4 that our proposed model achieves the best segmentation results of all tumors except for the fourth sample, where DenseUnet got the worst segmentation result with the highest degree of FP and the other three models provided a similar degree of precision. Besides, ResUnet segmentation result is the worst for the second sample, since it has mistaken some tumor areas as healthy tissues. For these four tumors, AAWS-Net properly classified the blue pixels (TP) and black pixels (TN), which illustrated that AAWS-Net could accurately distinguish between predicted object tags and ground truth masks. At the same time, very few green pixels (FP) indicate that the proposed AAWS-Net accurately classifies the unhealthy tissue as breast tumor area. Meanwhile, red pixels (FN) around the borders of tumor shows that AAWS-Net has failed to recognize a tiny area of tumor areas since healthy and unhealthy tissues are usually mixed together resulting in indiscernible borders of the tumor.

From the experimental results, it can be concluded that the proposed AAWS-Net is the most effective method for breast mass segmentation compared to other recently used semantic segmentation approaches.

### Ablation experiment results and discussion

In this section, an ablation study was conducted to comprehensively verify the rationality and the efficiency of AAWS-Net. In particular, we analyzed the results of the proposed AAWS-Net on CBIS-DDSM dataset with different network structures and different training strategies to understand the effects of anatomical structure and precise classification of breast mass on segmentation performance. Table 2 provides the segmentation metrics of seven models with different patterns, where Unet_Pre denotes the pre-trained Unet, AE_Pre represents the pre-trained auto-encoder, Without_KD is AAWS-Net without KD loss and Without_KL is AAWS-Net without KL loss.

**Table 2:** The segmentation metrics of ablation experiments.

Ablation Experiments	IoU	RE	F1
U-Net	0.628	0.684	0.738
Unet_Pre	0.634	0.718	0.754
AE	0.331	0.361	0.450
AE_Pre	0.391	0.412	0.528
Without_KD	0.659	0.748	0.773
Without_KL	0.655	0.746	0.775
AAWS-Net	**0.672**	**0.838**	**0.791**

We observed from Table 2 that AE achieved the worst segmentation result on all evaluation matrices, which indicates that AE is not suitable for semantic segmentation. In comparison with AE, the metrics of IoU, Recall and F1 score of pre-trained AE by reconstructing original images shows a remarkable improvement by 6%, 5.1% and 7.8%, respectively. Whereas, the degree of all metrics of pre-trained AE still the worst when compared to other models, which shows that without skip connections, the capability of anatomy features extraction would be weakened.

The traditional U-Net outperforms both AE and the pre-trained AE across all evaluation metrics, i.e., IoU, RE and F1, which are 62.8%, 68.4% and 73.8%, respectively. The improvements mainly benefit from symmetrical architecture with skip connections, whereas, the network only focused pixel-wise features rather than anatomical structures for per-pixel analysis, which constraints the upper limit for segmentation performance. Besides, the pre-trained U-Net consistently performs well on all evaluation metrics when contrasted to traditional U-Net, which means anatomical structures could assist the network in precisely discriminating breast mass from mammograms.

Now, we look at results from AAWS-Net when excluding KL loss. Compared with the pre-trained U-Net, classification of breast mass increases RE metric by 71.8% to 74.6%, which suggests that mass classification could provide discriminative clew information about masses existence to the network. Whereas, AAWS-Net still falls short of the state-of-the-art methods, due to the lack of intuitive information, i.e., anatomy features.

To figure out whether the U-Net can get any effective intuitive information from anatomical structures and to weigh anatomy features’ relative importance to segmentation results, we adopt KL loss but discard the KD loss when distilling the Student. Compared to the feature learning method which abandons KL loss, this strategy achieves higher value of IoU and RE of 65.9% and 74.8% respectively. Owing to intrinsic regularized anatomical structures in different mammographic images, we compare their common anatomy features, to guide the Student to segment breast masses, thus making the performance improved.

It is obvious that with the combination of KD loss and KL loss, the performance of AAWS-Net upgrades with the increase of 1.3%, 9%, and 1.8% for IoU, RE and F1, respectively. We can also see that solely adding KL loss or KD loss leads to a higher performance of our proposed model, where IoU, RE and F1 increase almost 3%, 4% and 4%, respectively, while the degree of all metrics of AAWS-Net with KL loss or KD loss has a tiny difference. It shows that the combination of tumor type classification and anatomy features is beneficial for promoting the sensitivity of the network to malignant masses, thus improving segmentation performance.

**Fig 5:**
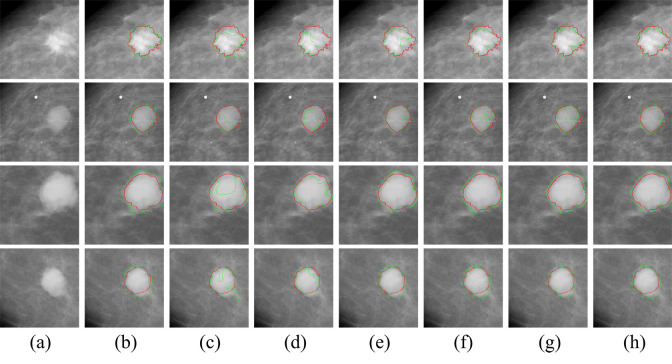
Segmentation results of seven models on CBIS-DDSM.

**Fig 6:**
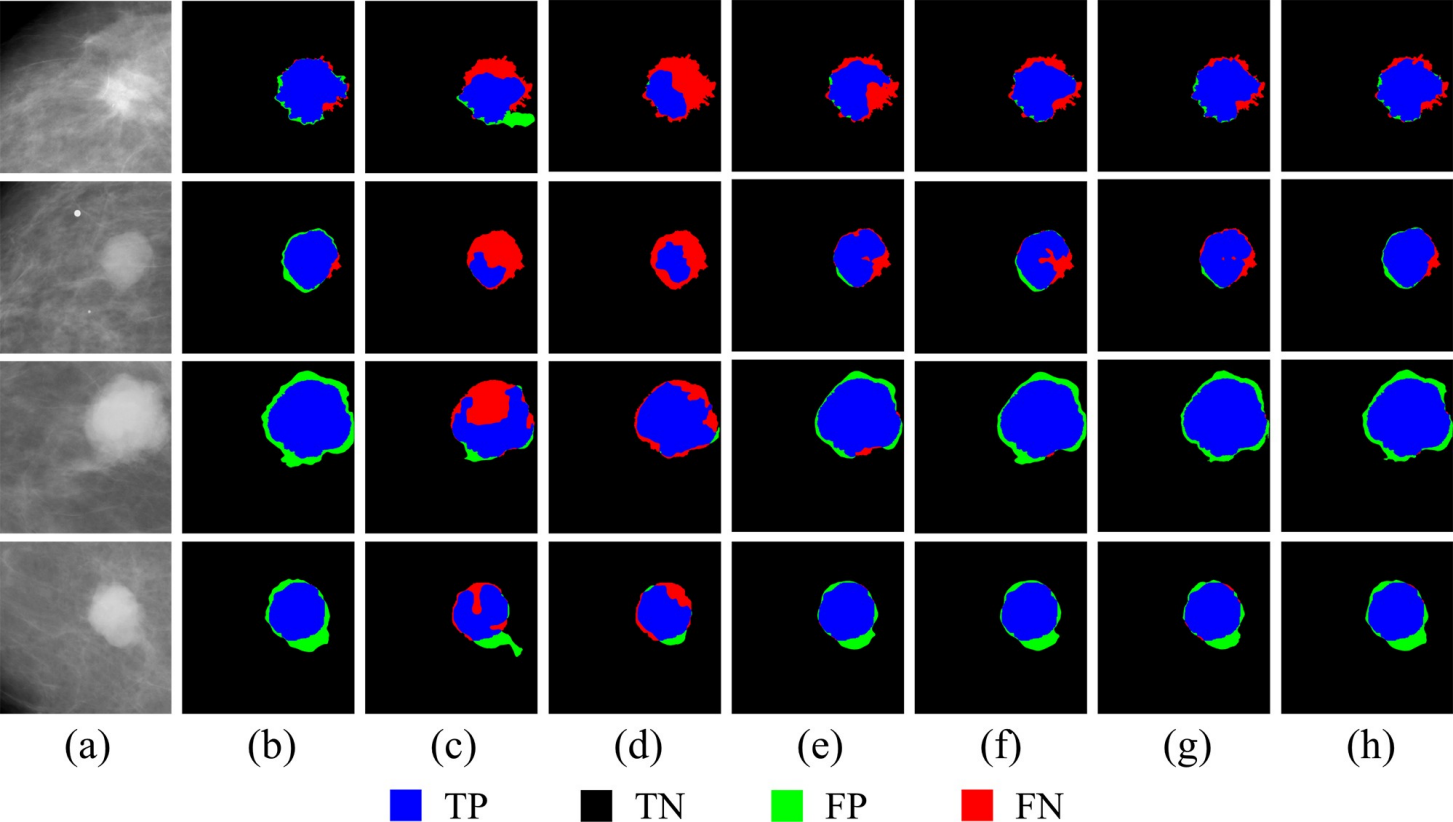
Segmentation results of seven models with four tumor samples.

Fig 5 shows the segmentation results of our method and other network models for four breast tumor samples, and Fig 6 is the visualization of the segmentation performance by our proposed method and other network models.

In Fig 5, the ground truth is shown in red, while the predicted tumor tags are denoted in green, and in Fig 6, the TP, TN, FP and FN are shown in blue, black, green and red, respectively. Both in Figs 5 and 6, column (a) and (b) are the original ROI images obtained from CBIS-DDSM, and column (c)-(h) are the segmentation results from AAWS-Net, AE, pre-trained AE, pre-trained U-Net, U-Net, AAWS-Net without KD loss, and AAWS-Net without KL loss, respectively. As we can see from Figs 5 and 6, AAWS-Net integrating KD loss and KL loss all provide the best results for all samples when compared to the others. Both AE and the pre-trained AE are under segmentation since they misclassify the tumor area as heathy tissues. Meanwhile, the pre-trained U-Net could perform fine contour detection and object segmentation than traditional U-Net, which indicates that anatomical structures have contributed to precisely outline the borders of breast mass. Moreover, for the first tumor, our proposal generates a predicted tumor’s edge which is irregular but more similar to the ground truth edges when compared to AAWS-Net without KD loss model and AAWS-Net without KL loss model. It shows that mass classification could provide discriminative clew information about masses’ existence to the network and anatomical structure could promote the sensitivity of the network to different masses.

In conclusion, the ablation experiments show that both anatomical structures and tumor classification are crucial for segmentation performance, and the architecture of our proposed AAWS-Net is reasonable and effective.

## Conclusion

In this study, considering the scarcity of image-level annotations of medical images, the under-utilization of numerous weakly-annotated data, and the under-exploration of anatomy features of breast, an Anatomy-Aware Weakly-Supervised Learning Network (AAWS-Net) was proposed for breast mass segmentation. In the proposed framework, AE was designed for extracting anatomy features from large-scale mammographic images with weak annotations, KD was adopted as a typical learning strategy to transfer prior knowledge to enhance segmentation performance, and KD loss was formulated for suggesting the morphological difference between benign and malignant mass. Besides, we compared AAWS-Net with four commonly used segmentation methods on CBIS-DDSM datasets and the experimental results showed the dominance of AAWS-Net across all evaluation metrics. Moreover, we also conducted an ablation study to evaluate the effectiveness of the two employed losses, namely KD loss and KL loss, and the ablation experiment’s results proved that both anatomy and morphological features can significantly improve the segmentation results, thus further improving the interpretability of AAWS-Net.

However, this study has the following limitations. First, due to the high resolution of X-ray images, the memory required for model training is relatively large, which may limit the practical application of the model. Second, a common problem existing in mammographic images is that, due to the low contrast and the similarity of the anatomical structure between masses and normal tissue in mammograms, it is difficult for AAWS-Net to detect the edge of mass precisely, which means AAWS-Net could wrongly identify lesions as healthy tissue and vice versa. Thirdly, the parameters of the machine are different when the data is obtained from different centers. This inconsistency changes the distribution of the image so that the same tissue in different centers will have slight gray differences. When the model encounters data that have not been seen during training, the anatomical features learned by the model could be disturbed to a certain extent.

## References

[1] Ghoncheh M, Pournamdar Z, Salehiniya H. Incidence and Mortality and Epidemiology of Breast Cancer in the World. Asian Pacific Journal of Cancer Prevention. 2016;17(S3):43–46. doi: 10.7314/apjcp.2016.17.s3.43 . 27165206

[2] Biswas SK, Mukherjee DP. Recognizing Architectural Distortion in Mammogram: A Multiscale Texture Modeling Approach with GMM. IEEE Transactions on Biomedical Engineering. 2011;58(7):2023–2030. doi: 10.1109/TBME.2011.2128870 . 21421429

[3] Abbas S, Jalil Z, Javed AR, Batool I, Khan M, Noorwali A, et al. BCD-WERT: a novel approach for breast cancer detection using whale optimization based efficient features and extremely randomized tree algorithm. PeerJ Computer Science. 2021;7:e390. doi: 10.7717/peerj-cs.390 33817036 PMC7959601

[4] Javed AR, Sarwar MU, Beg MO, Asim M, Baker T, Tawfik H. A collaborative healthcare framework for shared healthcare plan with ambient intelligence. Human-centric Computing and Information Sciences. 2020;10(1):40. 10.1186/s13673-020-00245-7.

[5] Silverstein MJ, Lagios MD, Recht A, Allred DC, Harms SE, Holland R, et al. Image-detected breast cancer: State of the art. diagnosis and treatment. Journal of the American College of Surgeons. 2005;201(4):586–597. doi: 10.1016/j.jamcollsurg.2005.05.032 . 16183499

[6] Di Maggio C. State of the art of current modalities for the diagnosis of breast lesions. European Journal of Nuclear Medicine and Molecular Imaging. 2004;31:S56–S69. doi: 10.1007/s00259-004-1527-8 . 15085294

[7] Elmore JG, Jackson SL, Abraham L, Miglioretti DL, Carney PA, Geller BM, et al. Variability in Interpretive Performance at Screening Mammography and Radiologists’ Characteristics Associated with Accuracy. Radiology. 2009;253(3):641–651. doi: 10.1148/radiol.2533082308 . 19864507 PMC2786197

[8] Gao Y, Geras KJ, Lewin AA, Moy L. New Frontiers: An Update on Computer-Aided Diagnosis for Breast Imaging in the Age of Artificial Intelligence. American Journal of Roentgenology. 2019;212(2):300–307. doi: 10.2214/AJR.18.20392 . 30667309 PMC6927034

[9] Taghanaki SA, Abhishek K, Cohen JP, Cohen-Adad J, Hamarneh G. Deep semantic segmentation of natural and medical images: a review. Artif Intell Rev. 2021;54(1):137–178. 10.1007/s10462-020-09854-1.

[10] Shen LZ, He MF, Shen N, Yousefi N, Wang C, Liu GQ. Optimal breast tumor diagnosis using discrete wavelet transform and deep belief network based on improved sunflower optimization method. Biomed Signal Process Control. 2020;60:10. 10.1016/j.bspc.2020.101953.

[11] Ahlem M. A Novel Region Growing Segmentation Algorithm for Mass Extraction in Mammograms. In: Amine A, Otmane AM, Bellatreche L, editors. Modeling Approaches and Algorithms for Advanced Computer Applications Studies in Computational Intelligence: Springer International Publishing; 2013. p. 95–104. doi: 10.1002/oby.20207

[12] Hu HZ. Segmentation of Breast Mass and Diagnosis o f Benign and Malignant Breast Tumors Based on Edge Constraint in Pulse Coupled Neural Network. J Med Imaging Health Inform. 2020;10(7):1597–1602. 10.1166/jmihi.2020.3086.

[13] Fu Y, Lei Y, Wang T, Curran WJ, Liu T, Yang X. A review of deep learning based methods for medical image multi-organ segmentation. Physica Medica. 2021;85:107–22. doi: 10.1016/j.ejmp.2021.05.003 33992856 PMC8217246

[14] Cireşan DC, Giusti A, Gambardella LM, Schmidhuber J. Deep neural networks segment neuronal membranes in electron microscopy images. Proceedings of the 25th International Conference on Neural Information Processing Systems; Lake Tahoe, Nevada: Curran Associates Inc.; 2012. p. 2843–2851. doi: 10.1016/j.neunet.2012.02.023

[15] Long J, Shelhamer E, Darrell T. Fully Convolutional Networks for Semantic Segmentation. IEEE Trans Pattern Anal Mach Intell; 2014. p. 640–651. doi: 10.1109/TPAMI.2016.2572683 . 27244717

[16] Badrinarayanan V, Kendall A, Cipolla R. SegNet: A Deep Convolutional Encoder-Decoder Architecture for Image Segmentation. IEEE Trans Pattern Anal Mach Intell. 2017;39(12):2481–2495. doi: 10.1109/TPAMI.2016.2644615 . 28060704

[17] Chen L-C, Zhu Y, Papandreou G, Schroff F, Adam H. Encoder-Decoder with Atrous Separable Convolution for Semantic Image Segmentation. In: Ferrari V, Hebert M, Sminchisescu C, Weiss Y, editors. Computer Vision–ECCV 2018: Springer International Publishing; 2018. p. 833–851. 10.1007/978-3-030-01234-2_49.

[18] Zhu W, Xiang X, Tran TD, Hager GD, Xie X. Adversarial deep structured nets for mass segmentation from mammograms. 2018 IEEE 15th International Symposium on Biomedical Imaging (ISBI); 2018. p. 847–850. https://doi.org/12.1109/ISBI.2018.8363704.

[19] Ronneberger O, Fischer P, Brox T. U-Net: Convolutional Networks for Biomedical Image Segmentation. In: Navab N, Hornegger J, Wells WM, Frangi AF, editors. Medical Image Computing and Computer-Assisted Intervention–MICCAI 2015: Springer International Publishing; 2015. p. 234–241. 10.1007/978-3-319-24574-4_28.

[20] Wang Z, Zou Y, Liu PX. Hybrid dilation and attention residual U-Net for medical image segmentation. Computers in Biology and Medicine. 2021;134:104449. doi: 10.1016/j.compbiomed.2021.104449 33993015

[21] Khanna A, Londhe ND, Gupta S, Semwal A. A deep Residual U-Net convolutional neural network for automated lung segmentation in computed tomography images. Biocybernetics and Biomedical Engineering. 2020;40(3):1314–1327. 10.1016/j.bbe.2020.07.007.

[22] Diakogiannis FI, Waldner F, Caccetta P, Wu C. ResUNet-a: A deep learning framework for semantic segmentation of remotely sensed data. ISPRS Journal of Photogrammetry and Remote Sensing. 2020;162:94–114. 10.1016/j.isprsjprs.2020.01.013.

[23] N RR R V, N E, Ramesh N. Deeply supervised U-Net for mass segmentation in digital mammograms. International Journal of Imaging Systems and Technology. 2021;31(1):59–71. 10.1002/ima.22516.

[24] Li S, Dong M, Du G, Mu X. Attention Dense-U-Net for Automatic Breast Mass Segmentation in Digital Mammogram. IEEE Access. 2019;7:59037–59047. 10.1109/ACCESS.2019.2914873.

[25] Zhang R, Zhang H, Chung ACS. A Unified Mammogram Analysis Method via Hybrid Deep Supervision. In: Stoyanov D, Taylor Z, Kainz B, Maicas G, Beichel RR, Martel A, et al., editors. Image Analysis for Moving Organ, Breast, and Thoracic Images: Springer International Publishing; 2018. p. 107–115. 10.1007/978-3-030-00946-5_12.

[26] Zhou Z, Rahman Siddiquee MM, Tajbakhsh N, Liang J. UNet++: A Nested U-Net Architecture for Medical Image Segmentation. In: Stoyanov D, Taylor Z, Carneiro G, Syeda-Mahmood T, Martel A, Maier-Hein L, et al., editors. Deep Learning in Medical Image Analysis and Multimodal Learning for Clinical Decision Support: Springer International Publishing; 2018. p. 3–11. 10.1007/978-3-030-00889-5_1. PMC7329239 32613207

[27] Zyuzin V, Chumarnaya T. Comparison of Unet architectures for segmentation of the left ventricle endocardial border on two-dimensional ultrasound images. 2019 Ural Symposium on Biomedical Engineering, Radioelectronics and Information Technology (USBEREIT); 2019. p. 110–113. 10.1109/USBEREIT.2019.8736616.

[28] Huang H, Lin L, Tong R, Hu H, Zhang Q, Iwamoto Y, et al. UNet 3+: A Full-Scale Connected UNet for Medical Image Segmentation. 2020 IEEE International Conference on Acoustics, Speech and Signal Processing (ICASSP); 2020. p. 1055–1059. 10.1109/icassp40776.2020.9053405.

[29] Tran S, Cheng C, Nguyen T, Le M, Liu D. TMD-Unet: Triple-Unet with Multi-Scale Input Features and Dense Skip Connection for Medical Image Segmentation. Healthcare. 2021;9(1):54. doi: 10.3390/healthcare9010054 33419018 PMC7825313

[30] Xie X, Chen J, Li Y, Shen L, Ma K, Zheng Y. Instance-Aware Self-supervised Learning for Nuclei Segmentation. In: Martel AL, Abolmaesumi P, Stoyanov D, Mateus D, Zuluaga MA, Zhou SK, et al., editors. Medical Image Computing and Computer Assisted Intervention–MICCAI 2020: Springer International Publishing; 2020. p. 341–350.

[31] Alialy R, Tavakkol S, Tavakkol E, Ghorbani-Aghbologhi A, Ghaffarieh A, Kim SH, et al. A Review on the Applications of Crowdsourcing in Human Pathology. J Pathol Inform. 2018;9:2–2. doi: 10.4103/jpi.jpi_65_17 29531847 PMC5841017

[32] Ouyang X, Xue Z, Zhan Y, Zhou XS, Wang Q, Zhou Y, et al. Weakly Supervised Segmentation Framework with Uncertainty: A Study on Pneumothorax Segmentation in Chest X-ray. In: Shen D, Liu T, Peters TM, Staib LH, Essert C, Zhou S, et al., editors. Medical Image Computing and Computer Assisted Intervention–MICCAI 2019: Springer International Publishing; 2019. p. 613–621. 10.1007/978-3-030-32226-7_68.

[33] Yang G, Wang C, Yang J, Chen Y, Tang L, Shao P, et al. Weakly-supervised convolutional neural networks of renal tumor segmentation in abdominal CTA images. BMC Medical Imaging. 2020;20(1):37. doi: 10.1186/s12880-020-00435-w 32293303 PMC7161012

[34] Girum KB, Créhange G, Hussain R, Lalande A. Fast interactive medical image segmentation with weakly supervised deep learning method. International Journal of Computer Assisted Radiology and Surgery. 2020;15(9):1437–1444. doi: 10.1007/s11548-020-02223-x 32653985

[35] Huang R, Zheng Y, Hu Z, Zhang S, Li H. Multi-organ Segmentation via Co-training Weight-Averaged Models from Few-Organ Datasets. In: Martel AL, Abolmaesumi P, Stoyanov D, Mateus D, Zuluaga MA, Zhou SK, et al., editors. Medical Image Computing and Computer Assisted Intervention–MICCAI 2020: Springer International Publishing; 2020. p. 146–155.

[36] Li K, Wang S, Yu L, Heng P-A. Dual-Teacher: Integrating Intra-domain and Inter-domain Teachers for Annotation-Efficient Cardiac Segmentation. In: Martel AL, Abolmaesumi P, Stoyanov D, Mateus D, Zuluaga MA, Zhou SK, et al., editors. Medical Image Computing and Computer Assisted Intervention–MICCAI 2020: Springer International Publishing; 2020. p. 418–427.

[37] Lu Y, Zheng K, Li W, Wang Y, Harrison AP, Lin C, et al. Contour Transformer Network for One-shot Segmentation of Anatomical Structures. IEEE transactions on medical imaging. 2020. doi: 10.1109/TMI.2020.3043375. 33290215

[38] Oktay O, Ferrante E, Kamnitsas K, Heinrich M, Bai W, Caballero J, et al. Anatomically Constrained Neural Networks (ACNNs): Application to Cardiac Image Enhancement and Segmentation. IEEE Transactions on Medical Imaging. 2018;37(2):384–395. doi: 10.1109/TMI.2017.2743464 . 28961105

[39] Ambellan F, Tack A, Ehlke M, Zachow S. Automated segmentation of knee bone and cartilage combining statistical shape knowledge and convolutional neural networks: Data from the Osteoarthritis Initiative. Medical Image Analysis. 2019;52:109–118. doi: 10.1016/j.media.2018.11.009 . 30529224

[40] Hall FM, Storella JM, Silverstone DZ, Wyshak G. Nonpalpable breast lesions: recommendations for biopsy based on suspicion of carcinoma at mammography. Radiology. 1988;167(2):353–358. doi: 10.1148/radiology.167.2.3282256 . 3282256

[41] Al-Shamlan H, El-Zaart A. Feature extraction values for breast cancer mammography images. 2010 International Conference on Bioinformatics and Biomedical Technology; 2010. p. 335–340. 10.1109/icbbt.2010.5478947.

[42] Hu M, Maillard M, Zhang Y, Ciceri T, La Barbera G, Bloch I, et al. Knowledge Distillation from Multi-modal to Mono-modal Segmentation Networks. In: Martel AL, Abolmaesumi P, Stoyanov D, Mateus D, Zuluaga MA, Zhou SK, et al., editors. Medical Image Computing and Computer Assisted Intervention–MICCAI 2020: Springer International Publishing; 2020. p. 772–781.

[43] Hu K, Zhang Z, Niu X, Zhang Y, Cao C, Xiao F, et al. Retinal vessel segmentation of color fundus images using multiscale convolutional neural network with an improved cross-entropy loss function. Neurocomputing. 2018;309:179–191. 10.1016/j.neucom.2018.05.011.

[44] Bowyer K, Kopans D, Kegelmeyer WP. The digital database for screening mammography. Image Analysis for Moving Organ, Breast, and Thoracic Images: Springer International Publishing. 1996.

[45] Lee RS, Gimenez F, Hoogi A, Miyake KK, Gorovoy M, Rubin DL. A curated mammography data set for use in computer-aided detection and diagnosis research. Scientific Data. 2017;4. doi: 10.1038/sdata.2017.177. 29257132 PMC5735920

[46] Guan S, Khan AA, Sikdar S, Chitnis PV. Fully Dense UNet for 2-D Sparse Photoacoustic Tomography Artifact Removal. IEEE Journal of Biomedical and Health Informatics. 2020;24(2):568–576. doi: 10.1109/JBHI.2019.2912935 . 31021809

[47] Girshick R, Donahue J, Darrell T, Malik J. Rich Feature Hierarchies for Accurate Object Detection and Semantic Segmentation. 2014 IEEE Conference on Computer Vision and Pattern Recognition; 2014. p. 580–587.

